# Modified thoracoabdominal nerve block via perichondral approach: an alternative for perioperative pain management in laparoscopic cholecystectomy in a middle-income country

**DOI:** 10.1186/s12871-024-02690-8

**Published:** 2024-08-31

**Authors:** Luisa Fernanda Castillo-Dávila, Carlos Jesús Torres-Anaya, Raquel Vazquez-Apodaca, Hector Borboa-Olivares, Salvador Espino-y-Sosa, Johnatan Torres-Torres

**Affiliations:** 1 Anesthesiology Department, Hospital General de México Dr. Eduardo Liceaga, Mexico City, Mexico; 2 Community Interventions Research Branch, Instituto Nacional de Perinatología “Isidro Espinosa de los Reyes”, Mexico City, Mexico; 3 Department of Reproductive and Perinatal Health Research, Instituto Nacional de Perinatología Isidro Espinosa de los Reyes, Mexico City, Mexico

**Keywords:** Laparoscopic cholecystectomy, Modified thoracoabdominal nerve block (M-TAPA), Perioperative pain management, Opioid consumption, Postoperative analgesia

## Abstract

**Background:**

Laparoscopic cholecystectomy is known for its minimally invasive nature, but postoperative pain management remains challenging. Despite the enhanced recovery after surgery (ERAS) protocol, regional analgesic techniques like modified perichondral approach to thoracoabdominal nerve block (M-TAPA) show promise. Our retrospective study evaluates M-TAPA’s efficacy in postoperative pain control for laparoscopic cholecystectomy in a middle-income country.

**Methods:**

This was a retrospective case-control study of laparoscopic cholecystectomy patients at Hospital General de Mexico in which patients were allocated to the M-TAPA or control group. The data included demographic information, intraoperative variables, and postoperative pain scores. M-TAPA blocks were administered presurgery. Outcomes: opioid consumption, pain intensity, adverse effects, and time to rescue analgesia. Analysis of variance (ANOVA) compared total opioid consumption between groups, while Student’s t test compared pain intensity and time until the first request for rescue analgesia.

**Results:**

Among the 56 patients, those in the M-TAPA group had longer surgical and anesthetic times (*p* < 0.001), higher ASA 3 scores (25% vs. 3.12%, *p* = 0.010), and reduced opioid consumption (*p* < 0.001). The M-TAPA group exhibited lower postoperative pain scores (*p* < 0.001), a lower need for rescue analgesia (*p* = 0.010), and a lower incidence of nausea/vomiting (*p* = 0.010).

**Conclusion:**

Bilateral M-TAPA offers effective postoperative pain control after laparoscopic cholecystectomy, especially in middle-income countries, by reducing opioid use and enhancing recovery.

## Introduction

Laparoscopic cholecystectomy is a cornerstone procedure in modern surgery and is renowned for its minimally invasive approach and swift recovery compared to open surgery [1]. However, managing postoperative pain, which can range from moderate to severe within the first 24 h, remains a clinical challenge [2]. This pain, driven primarily by somatic components followed by neuropathic and shoulder-referred pain, exhibits considerable variability among patients [3].

To address this challenge, the enhanced recovery after surgery (ERAS) protocol advocates for a multimodal analgesic strategy [4]. However, the efficacy of nonopioid analgesics and adjuvant anesthetics has limitations, spurring exploration of regional analgesic techniques such as ultrasound-guided nerve blocks or local infiltration [5, 6].

By targeting the anterolateral abdominal wall innervated by the thoracoabdominal nerves, the modified perichondral approach to thoracoabdominal nerve block (M-TAPA) has emerged as a promising analgesic modality [7–9]. M-TAPA, introduced by Tulgar et al. in 2019, offers broader coverage of both anterior and lateral cutaneous branches of the thoracoabdominal nerves, surpassing the limitations of conventional techniques such as the transversus abdominis plane block [10]. While initial studies have demonstrated its efficacy in abdominal surgeries, including laparoscopic cholecystectomy, further research is needed to establish its clinical utility and compare it with existing regional analgesic methods [11, 12].

Healthcare in middle-income countries faces considerable challenges due to resource limitations and underdeveloped healthcare infrastructure. These nations often lack access to advanced treatments and pain management techniques, underscoring the importance of developing effective and economically viable approaches to address perioperative pain. In this context, our study aimed to assess the efficacy of M-TAPA for postoperative pain management compared to that of conventional analgesia in laparoscopic cholecystectomy patients in a middle-income country.

## Methods

### Study design and participants

This retrospective case-control study involved adult patients who underwent laparoscopic cholecystectomy at the Hospital General de Mexico “Dr. Eduardo Liceaga” in Mexico City from January 2023 to July 2023. Patients were retrospectively categorized into two groups based on the analgesic technique they received during surgery: those who received M-TAPA were assigned to the M-TAPA group, while those who received local infiltration were allocated to the control group. The study was conducted with the approval of the institutional research and bioethics boards at the Hospital General de Mexico “Dr. Eduardo Liceaga” (Approval number: 1384 − 295/23). Exclusion criteria included allergies to local anesthetics or contraindications to nerve block procedures.

### Data collection

The data, including demographic information; intraoperative variables such as surgical and anesthetic duration; intraoperative opioid consumption; and postoperative pain scores assessed using the visual analog scale (VAS) [13, 14], which ranges from 0 to 10, at awake, 30, and 120 min postoperatively, were extracted from the patients’ medical records. Each of these variables of interest was then transferred to an electronic database for analysis. Adverse effects related to analgesia, such as nausea and vomiting, were also recorded. Furthermore, our analysis incorporated variables such as time to mobilization and time to complete oral intake to assess postoperative recovery. Additionally, the time until the first request for rescue analgesia was documented as a measure of postoperative pain management efficacy.

### Anesthesia and perioperative management protocol

No preoperative premedication was administered to the patients. The anesthesia technique was standardized for all patients in the operating room, involving electrocardiography, non-invasive blood pressure monitoring, capnography, peripheral oxygen saturation, neuromonitoring, a multi-gas analyzer, and temperature monitoring, alongside the initiation of a 0.9% NaCl infusion at a rate of 4 ml/kg.

Anesthesia induction comprised propofol (1.2 mg/kg), rocuronium (0.6 mg/kg), and fentanyl (4 mcg/kg) based on ideal body weight. Following endotracheal intubation, maintenance anesthesia involved a blend of 2% sevoflurane and 50% air in 50% O2 (3.5 L/min). Mechanical ventilation was executed in volume-controlled mode, with tidal volume set at 6–8 ml/kg according to ideal body weight to maintain end-tidal carbon dioxide at 30 to 35 mm Hg.

During surgery, anesthesia depth was regulated using end-tidal sevoflurane, maintaining sevoflurane concentration at 0.8-1 MAC.

In the M-TAPA group, the regional blockade was bilaterally administered by a single anesthesiologist post-general anesthesia induction and pre-surgical procedure. Sevoflurane maintained anesthesia and in most cases as heart rate and blood pressure values remained within a 20% variation, there was no need for additional boluses of fentanyl (1 mcg/kg).

In the control group, intraoperative management involved fentanyl infusion titration, supplemented in some cases with dexmedetomidine or lidocaine infusion, while ensuring non-invasive blood pressure and heart rate remained within 20% variation.

Patients were transferred to the post-anesthesia care unit upon completion of surgery, once neuromuscular functions were fully restored, and adequate tidal volume was observed.

Standard analgesia comprised intravenous administration of paracetamol 1 g every 8 h, metamizole 1 g every 8 h, or ketorolac 60 mg every 8 h. The initial doses of paracetamol and metamizol or ketorolac were administered in the last 30 min of surgery. Additionally, 100 mg of tramadol was intravenously administered to all patients in the no-block group before surgery termination. Pain intensity was assessed using a 0–10 numeric rating scale (NRS) at rest and during movement. Tramadol 50–100 mg was administered intravenously as a rescue analgesic to patients with a resting NRS > 4.

### Description of the M-TAPA block technique

After administering anesthesia and before surgery began, an experienced anesthesiologist performed the M-TAPA block while the patient laid on their back. Using a high-frequency linear transducer (7–14 MHz, SonoSite M-Turbo), the anesthesiologist located the external, internal, and transverse abdominal muscles near the tenth rib cartilage, aiming to clearly visualize the underside of the cartilage.

Once the injection site was prepped, a 22G x 100 mm peripheral nerve block needle (Stimuplex^®^ Ultra 360^®^, B-Braun, USA) was carefully guided toward the underside of the tenth rib cartilage between the internal oblique and transverse abdominal muscles using real-time ultrasound imaging. After confirming the correct placement of the saline solution and ensuring that there was no blood aspiration, 20 ml of 0.375% ropivacaine was injected, ensuring effective anesthetic spread between the muscle layers. The same process was performed bilaterally. All blocks were conducted by an experienced anesthesiologist [12, 15].

### Outcome

The primary outcome measure was total opioid consumption during the first postoperative hour. The secondary outcomes included postoperative pain intensity, incidence of adverse effects related to analgesia, and time to the first request for rescue analgesia.

### Statistical analysis

Continuous variables are expressed as medians and interquartile ranges (IQRs), and inferential testing was performed using the Mann‒Whitney U test; categorical data are expressed as numbers and percentages and were analyzed using the chi‒square test or Fisher’s exact test. Analysis of variance (ANOVA) was used to compare total opioid consumption between groups, and Student’s t test was used to compare pain intensity and time until the first request for rescue analgesia. A *p*-value of less than 0.05 was considered to indicate statistical significance. (StataCorp. 2020, Stata Statistical Software: Release 17. College Station, TX: StataCorp LLC).

## Results

### Characteristics of the study population

A total of 56 patients were analyzed in this study, with a predominance of 69.6% females and 30.4% males. The mean age of the patients was 38.18 ± 9.57 years. Clinical characteristics such as age, sex, and body mass index were significantly similar between the study groups **(**Table 1**)**. However, compared with those in the control group, a significantly greater proportion of patients in the M-TAPA group underwent ASA 3 assessment (25% vs. 3.12%, *p* = 0.010).

**Table 1:** Characteristics of the study population

Characteristic	M-TAPA group (*n* = 24)	Control group (*n* = 32)	*p* value
Age (years)	38.45 (9.06)	37.96 (10.07)	0.518
Gender			
Female	14 (58.33%)	25 (78.12%)	0.111
Male	10 (41.67%)	7 (21.88%)	
BMI	26.14 (3.09)	25.77 (3.10)	0.660
ASA			
1	3 (12.5%)	13 (40.62%)	0.010
2	15 (62.5%)	18 (56.25%)	
3	6 (25%)	1 (3.12%)	
Surgical time (min)	119.75 (41.15)	81.25 (14.25)	0.0004
Anesthetic time (min)	153.25 (51.81)	107.06 (14.49)	0.0001
Anesthetic adjuvants (%)	2 (8.33%)	25 (78.12%)	0.0001
Opioid consumption (mg)	341.58 (71.83)	577.53 (147.53)	0.0001

M-TAPA: modified perichondral approach to thoracoabdominal nerve block; BMI: body mass index.

### Characteristics of the surgical event

**Fig. 1:**
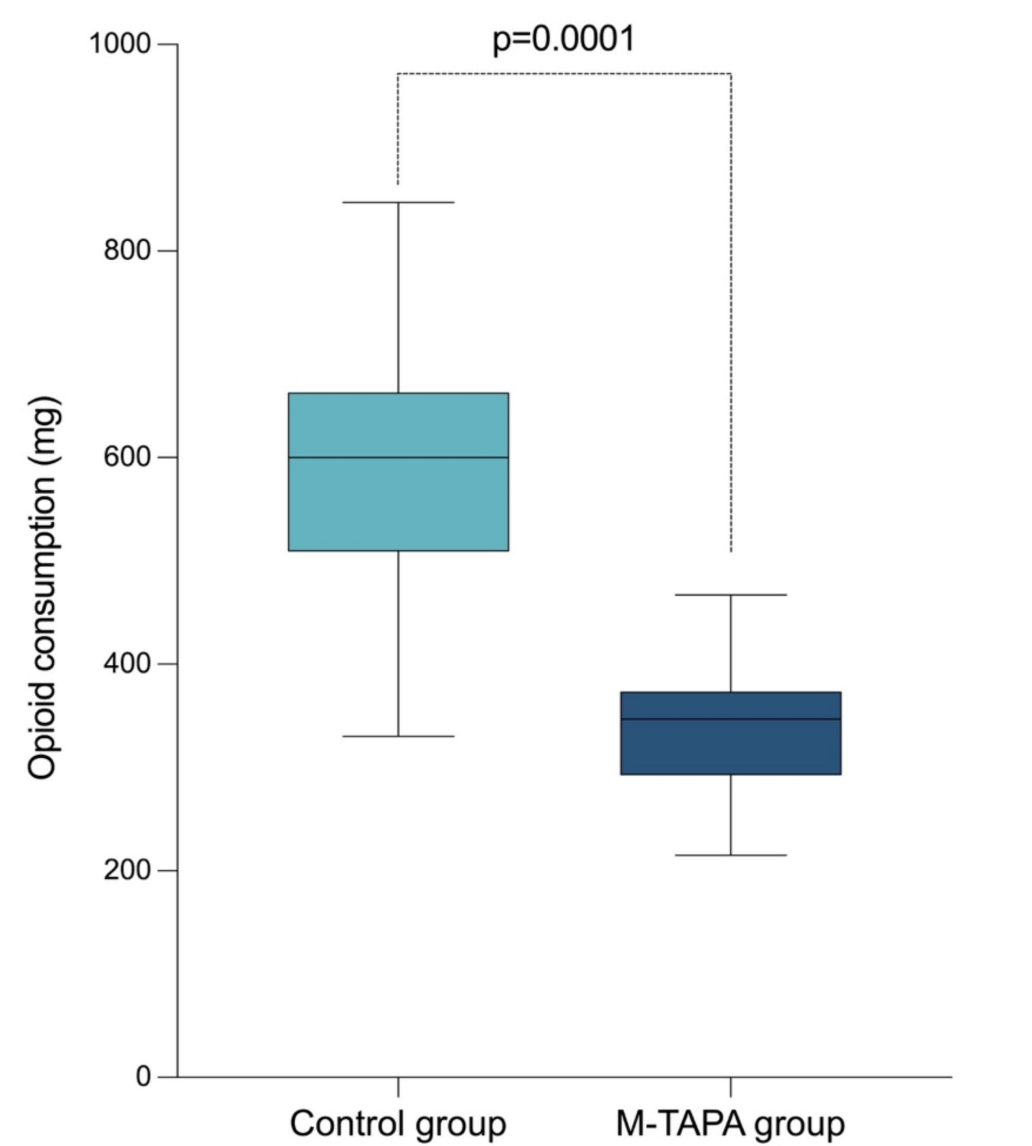
Opioid consumption between groups

Surgical and anesthetic times were significantly longer in the M-TAPA group than in the control group (119.75 *versus* 81.25, *p* = 0.0004 and 153.25 *versus* 107.06, *p* = 0.0001, respectively). Additionally, there was a greater use of anesthetic adjuvants in the control group (8.33% *versus* 78.12%, *p* = 0.0001), as did increased opioid consumption (341.58 *versus* 577.53, *p* = 0.0001) **(**Tables 1**and** Fig. 1**)**.

### Postoperative evaluation between groups

**Fig. 2:**
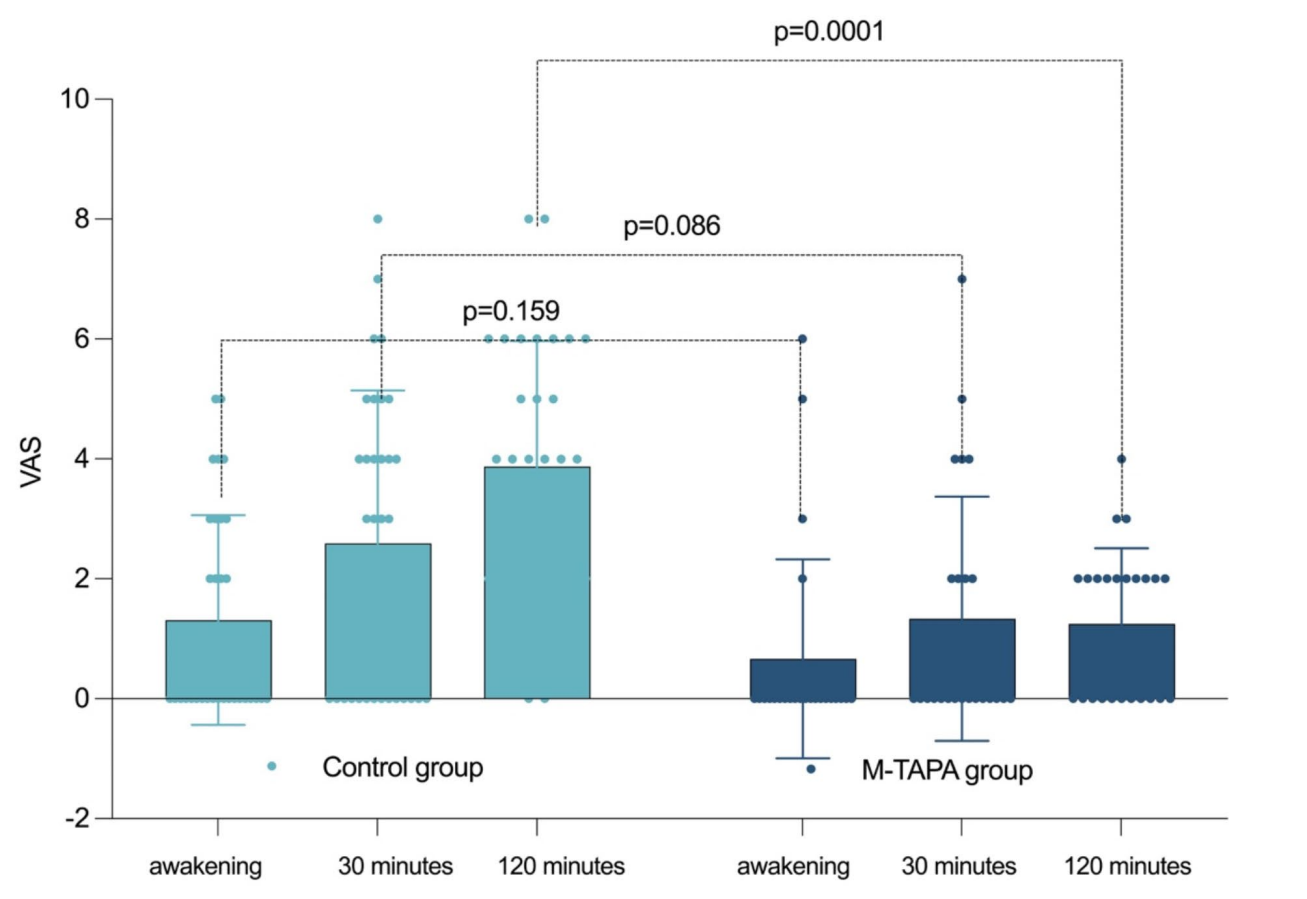
Postoperative pain assessment in the recovery area

**Fig. 3:**
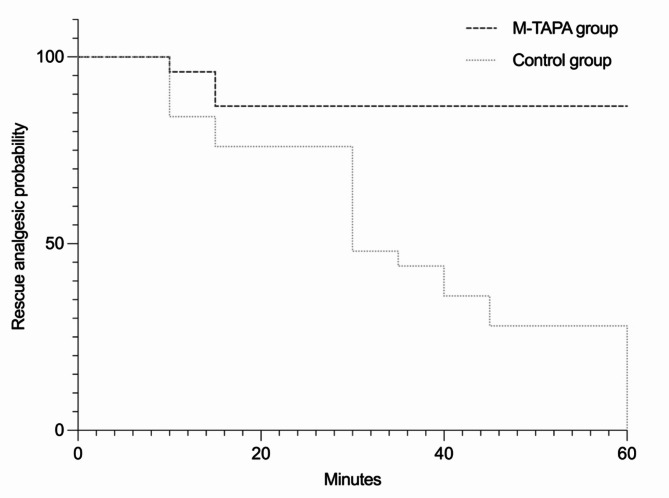
Depicts a Kaplan-Meier survival curve illustrating the time to rescue analgesia following laparoscopic cholecystectomy, with a focus on the need for tramadol rescue analgesia at 60 min post-surgery

There were no significant differences in the VAS scores at awakening or 30 min after the operation between the study groups **(**Table 2**)**. However, upon recovery, the M-TAPA group exhibited significantly lower pain scores than did the control group (1.25 *versus* 3.88, *p* = 0.0001) **(**Fig. 2**)**. In Fig. 3, the Kaplan-Meier survival curve compares the probability of patients in the control group and the M-TAPA group requiring tramadol rescue analgesia over time post-surgery. The log-rank test indicated a statistically significant difference between the survival curves (χ² =21.30, df = 1, *p* < 0.0001), demonstrating a longer duration until rescue analgesia in the M-TAPA group compared to the control group. This finding underscores the efficacy of M-TAPA in providing prolonged pain relief following laparoscopic cholecystectomy. Additionally, the hazard ratio (HR) calculated using the Mantel-Haenszel method was 8.333 (95%CI: 3.973 to 17.48), indicating a significantly lower risk of requiring rescue analgesia in the M-TAPA group compared to the control group. Furthermore, consistent with these survival analysis results, there was a reduced need for rescue analgesia with tramadol at 60 min post-surgery in the M-TAPA group compared to the control group (16.67% *versus* 50%, *p* = 0.010). A greater incidence of nausea and/or vomiting was recorded in the control group than in the M-TAPA group (15.62% *versus* 0, *p* = 0.010) **(**Table 2**)**. Furthermore, analysis revealed that the M-TAPA group had significantly shorter times for early mobilization (8.2 h *versus* 10.6 h, *p* = 0.010), and quicker resumption of oral intake (17.3 h *versus* 24.7 h, *p* = 0.001) compared to the control group.

**Table 2:** Postoperative evaluation

Characteristic	Block group (*n* = 24)	Control group (*n* = 32)	*p* value
VAS at awakening	0.7 (1.7)	1.3 (1.8)	0.159
VAS at 30 min	1.3 (2.0)	2.6 (2.6)	0.086
VAS at 120 min	1.3 (1.3)	3.9 (2.1)	0.0001
Rescue Analgesia	4 (16.67%)	16 (50%)	0.010
Tramadol Dose			
50 mg	2 (8.33%)	8 (25%)	0.036
100 mg	2 (8.33%)	8 (25%)	
Nausea and/or Vomiting	0	5 (15.62%)	0.010
Time to mobilization (hours)	8.2 (2.1)	10.6 (3.5)	0.010
Time to complete oral intake (hours)	17.3 (4.2)	24.7 (5.9)	0.001

VAS: visual analog scale

## Discussion

The findings of our study underscore the efficacy of the M-TAPA block in alleviating postoperative pain following laparoscopic cholecystectomy. While the analgesic effectiveness of this technique is independent of the socioeconomic context, conducting the study in a middle-income country enhances the universal applicability and external validity of the M-TAPA block. It demonstrates that this approach can be particularly beneficial in settings with limited resources, where cost-effective and easily implementable pain management strategies are crucial. In our study, the administration of M-TAPA post anesthesia led to a significant reduction in postoperative pain levels, decreased opioid consumption, and a lower requirement for rescue analgesia during the recovery period. These outcomes not only highlight the effectiveness of M-TAPA in improving patient comfort but also emphasize its potential as a valuable tool in resource-limited environments.

This study contributes to the growing body of evidence supporting the use of M-TAPA in abdominal surgeries. In line with the findings of Bilge et al. and Güngör et al. [11, 12]. , we found that M-TAPA provides effective analgesia, evidenced by a significant reduction in the need for rescue analgesia and lower postoperative pain scores compared to control groups receiving alternative analgesic approaches. Additionally, we observed a higher proportion of ASA 3 patients in the M-TAPA group, suggesting that this technique may be particularly beneficial for patients with more complex physical statuses.

Our results also align with Tulgar et al.‘s research [15], which underscores M-TAPA’s efficacy in abdominal surgeries, supporting our own observation of decreased opioid consumption and improved patient satisfaction. Furthermore, our study aligns with Chen et al.‘s findings, emphasizing the versatility of M-TAPA, as we illustrate its potential efficacy, even in obese patients [16].

The cadaveric studies by Ciftci et al. and the clinical cases presented by Aikawa et al. provide a robust anatomical and clinical basis for M-TAPA efficacy [17, 18]. Our findings support this understanding by demonstrating a reduced need for rescue analgesia, lower opioid consumption, and better postoperative recovery in the M-TAPA group compared to the control group. Our study did not directly assess the impact on recovery using instruments like the QoR-40 questionnaire employed in Bilge et al.‘s study or the QoR-15 questionnaire used in Suzuka et al.‘s research [12, 19]. Nonetheless, we have emphasized the potential for improved recovery based on objective metrics, such as notably shorter durations for early mobilization and faster resumption of oral intake observed in the M-TAPA group compared to the control group. These specific findings regarding functional aspects of recovery, namely mobilization and oral intake, serve as objective indicators suggesting that the M-TAPA technique may contribute to a swifter and smoother recovery process following laparoscopic cholecystectomy.

The consistent findings across several studies, including our own, underscore the reliability and practicality of this technique for postoperative pain management in abdominal surgeries.

### Strengths and limitations

Our study utilized objective and quantitative measures, such as intraoperative opioid consumption and postoperative pain intensity assessed using the visual analog scale, thereby enhancing the reliability and validity of our findings. The inclusion of demographic and clinical variables also allowed for a more comprehensive analysis and appropriate comparison between study groups.

However, several limitations must be acknowledged. The sample size and retrospective design of the present study may introduce selection and confounding biases, potentially compromising its internal validity. Uncontrolled factors such as variations in surgical technique, team experience, and anesthesiologist expertise could influence outcomes. Furthermore, long-term follow-up data for assessing surgical complications and late-stage recovery were lacking. The single-center design limits generalizability, and the absence of definitive recommendations on the optimal LA concentration underscores the need for prospective studies. Despite these limitations, our findings strongly support the efficacy of M-TAPA blockade for perioperative pain management, warranting further multicenter investigations.

### Clinical implications

Our study findings suggest that integrating M-TAPA into standard anesthesia protocols for laparoscopic cholecystectomy, particularly in a middle-income country setting, can notably enhance pain management outcomes. By decreasing intraoperative opioid usage and postoperative pain intensity, M-TAPA represents a promising strategy for improving patient comfort, safety, and overall surgical experience. This approach not only helps mitigate opioid-related side effects but also holds potential for expediting recovery, reducing hospital stays, and optimizing resource utilization in healthcare settings. These results highlight the importance of developing accessible and effective pain management strategies in resource-constrained environments, underscoring the need for greater consideration of middle-income countries in research and clinical practice related to perioperative pain management to improve outcomes and enhance the quality of care in these challenging settings.

## Conclusion

Our study highlights the effectiveness of M-TAPA for managing postoperative pain after laparoscopic cholecystectomy, especially in middle-income countries. By reducing opioid use and providing prolonged analgesia, MWA offers a safer alternative to traditional methods, potentially leading to the transformation of perioperative care. However, further research is needed to validate its broad applicability in diverse surgical settings.

## Data Availability

The datasets used and/or analyzed during the current study are available upon reasonable request. Access to the data can be requested via the following Google Drive link: https://drive.google.com/file/d/1uQWopPm2vmltwrh52_TKzjEvL1Z_Cqu-/view? usp=drivesdk. Please contact luisacast.259@gmail.com or torresmmf@gmail.com to request access.
